# Tumor suppressor PTEN affects tau phosphorylation: deficiency in the phosphatase activity of PTEN increases aggregation of an FTDP-17 mutant Tau

**DOI:** 10.1186/1750-1326-1-7

**Published:** 2006-07-31

**Authors:** Xue Zhang, Yun-wu Zhang, Shijie Liu, Ayelen Bulloj, Gary G Tong, Zhuohua Zhang, Francesca-Fang Liao, Huaxi Xu

**Affiliations:** 1 Center for Neuroscience and Aging, Burnham Institute for Medical Research, 10901 N. Torrey Pines Road, La Jolla, CA 92037, USA

## Abstract

**Background:**

Aberrant hyperphosphorylation of tau protein has been implicated in a variety of neurodegenerative disorders. Although a number of protein kinases have been shown to phosphorylate tau *in vitro *and *in vivo*, the molecular mechanisms by which tau phosphorylation is regulated pathophysiologically are largely unknown. Recently, a growing body of evidence suggests a link between tau phosphorylation and PI3K signaling. In this study, phosphorylation, aggregation and binding to the microtubule of a mutant frontal temporal dementia and parkinsonism linked to chromosome 17 (FTDP-17) tau in the presence of tumor suppressor PTEN, a major regulatory component in PI3K signaling, were investigated.

**Results:**

Phosphorylation of the human mutant FTDP-17 tau, T40RW, was evaluated using different phospho-tau specific antibodies in the presence of human wild-type or phosphatase activity null mutant PTEN. Among the evaluated phosphorylation sites, the levels of Ser214 and Thr212 phospho-tau proteins were significantly decreased in the presence of wild-type PTEN, and significantly increased when the phosphatase activity null mutant PTEN was ectopically expressed. Fractionation of the mutant tau transfected cells revealed a significantly increased level of soluble tau in cytosol when wild-type PTEN was expressed, and an elevated level of SDS-soluble tau aggregates in the presence of the mutant PTEN. In addition, the filter/trap assays detected more SDS-insoluble mutant tau aggregates in the cells overexpressing the mutant PTEN compared to those in the cells overexpressing wild-type PTEN and control DNA. This notion was confirmed by the immunocytochemical experiment which demonstrated that the overexpression of the phosphatase activity null mutant PTEN caused the mutant tau to form aggregates in the COS-7 cells.

**Conclusion:**

Tumor suppressor PTEN can alleviate the phosporylation of the mutant FTDP-17 tau at specific sites, and the phosphatase activity null PTEN increases the mutant tau phosphorylation at these sites. The changes of the tau phosphorylation status by ectopic expression of PTEN correlate to the alteration of the mutant tau's cellular distribution. In addition, the overexpression of the mutant PTEN can increase the level of the mutant tau aggregates and lead to the formation of visible aggregates in the cells.

## Background

Tauopathies, including Alzheimer's disease (AD), Pick's disease (PiD), corticobasal degeneration (CBD), progressive supranuclear palsy (PSP), argyrophilic grain disease and frontotemporal dementia and parkinsonism linked to chromosome 17 (FTDP-17), are a group of neurodegenerative disorders that are pathologically featured by intracellular neurofibrillary tangles (NFTs) [1,2]. Although the causal role of NFTs in neurodegeneration of tauopathies is still questionable, for example, the neurons with NFTs can live for years [3], and the mutations of amyloid precursor protein (APP) [4] and presenilins [5] are accused of the pathogenesis of AD, the neuronal toxicity of NFTs have been implicated by a number of studies in cellular and animal tauopathy models [2].

The major component of NFTs is bundles of paired helical filaments (PHF) of abnormally hyperphosphorylated tau proteins [6]. Tau is a class of microtubule-associated protein (MAP). The tau proteins are normally expressed in neuronal and glial cytoplasm including cell bodies, neurites and axons, where they bind to and stabilize microtubules [7-9]. Under normal physiological conditions, tau is phosphorylated at 2–3 serine and threonine sites before proline. *In vitro *studies have identified several proline-directed kinases that can phosphorylate tau at different sites, including cyclin-dependent kinase 5 (CDK5) [10], glycogen synthase kinase-3 (GSK-3) [11], mitogen-activated protein kinase (MAPK) [12,13], protein kinase A [14], protein kinase (PKC) [15,16] and Akt/protein kinase B (PKB) [17]. In tauopathies, tau is aberrantly hyperphosphorylated, carrying 3–4 times more phosphates [18,19]. The hyperphosphorylation of tau has been accused of causing tau dysfunction, aggregation, and likely NFT formation [20,21]. The evidence for a causal role of abnormal tau phosphorylation and aggregation in neurodegenerative disorders was supported by the genetic analyses of the inherited FTDP-17, which led to identification of tau FTDP-17 mutations that cause the disease [22-24]. However, the molecular mechanisms by which phosphorylation of tau protein is regulated pathophysiologically are largely unknown.

Recent studies have revealed aberrant upregulation of neuronal markers for mitogenic signaling pathways in the brains of tauopathy animals and AD patients. They include Akt and the target of rapamycin (TOR) that are downstream effectors of the tumor suppressor phosphatase and tensin homologue deleted on chromosome ten (PTEN)-regulated phosphoinositide-3 kinase (PI3K) signaling pathway, implying a link between PI3K signaling pathway and pathogenesis of AD and tauopathies [25-28]. In the PI3K signaling pathway, tumor suppressor PTEN antagonizes PI3K by dephosphorylating phosphatidylinositol (3,4,5)-triphosphate (PIP3) to regulate a variety of crucial cellular functions, including cell proliferation, migration and apoptosis [29,30].

The tumor suppressor gene *Pten*, also known as *MMAC1 *and *TEP1*, has been found to be mutated in many human sporadic and hereditary cancers [31-34]. Although PTEN exhibits both protein and lipid phosphatase activity *in vitro *[35], only PIP3 has been identified as a major lipid substrate for PTEN *in vivo *[35,36], leaving PTEN's protein substrate(s) unknown. Multiple lines of evidence from PTEN-null animal models have shown that PTEN is required for normal embryonic development [37-40] and that conditional inactivation of PTEN in the brain led to abnormal development of neurons [41,42]. Recently Griffin *et al*. showed decreased levels and altered distribution of PTEN along with elevated PI3K signaling in the brain of AD patients [25]. We also showed that overexpression of PTEN can affect phosphorylation of wild-type human tau at multiple sites, decrease tau aggregation and improve tau binding to microtubules in cells [43]. Given that tau phosphorylation is harmful to neurons, these results suggest that PTEN regulates tau phosphorylation through PI3K signaling and that the loss of PTEN functions may contribute to neurodegeneration in AD.

In the present study, in order to investigate whether PTEN can affect the phosphorylation, aggregation and microtubule binding ability of mutant tau associated with tauopathy, we used an FTDP-17 missense mutant tau, R406W, which has been shown to be less soluble and less capable of binding to microtubules than wild-type tau [44,45]. Here we demonstrate that PTEN inhibits tau phosphorylation at Akt sites, hence reducing the aggregation and promoting the binding to microtubules of an FTDP-17 mutant tau.

## Results

### Overexpression of PTEN affects the FTDP-17 mutant tau phosphorylation

**Figure 1:**
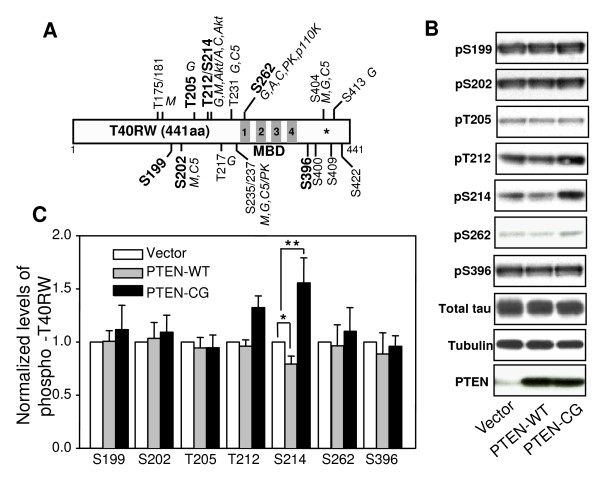
The effects of PTEN on phosphorylation of the FTDP-17 mutant tau. A. Schematic drawing of the FTDP-17 mutant tau, T40RW, and major phosphorylation sites by various kinases. The missense mutation of Arg to Trp at position 406 is labeled by a star. The examined phosphorylation sites are in bold. Kinases are abbreviated as follows: M, MAPK; G, GSK-3; C5, CDK5; A, PKA; C, PKC; PK, phosphorylase kinase. The four repeats of microtubule-binding domain (MBD) are shown as the filled areas. B. Vector, wild-type (PTEN-WT) or the phosphatase null mutant PTEN (PTEN-CG) transfected COS-7 cells were analyzed by Western blotting using phospho-tau specific antibodies as indicated. C. Levels of phospho-tau were quantified and normalized to the total tau level. Error bars indicate means ± SE, *n *= 4, *p < 0.02 and **p < 0.05.

Tau can be phosphorylated at multiple sites by various kinases. In a previous study, we found that tumor suppressor PTEN can affect wild-type human tau phosphorylation at several sites, including two Akt sites, Ser214 and Thr212. To further study whether PTEN affects phosphorylation and hence aggregation and microtubule association of FTDP-17 mutant tau proteins, we cotransfected the T40RW, a tau mutant identified from FTDP-17 [46], and human wild-type PTEN (PTEN-WT) or a mutant PTEN lacking the phosphatase activity (PTEN-CG) [47,48] into COS-7 cells. Using phospho-tau specific antibodies [49], we have evaluated the FTDP-17 mutant tau phosphorylation status at 7 different sites, including Akt targets, Thr212 and Ser214, GSK-3 targets, Ser199, Thr205 and Ser396, and two paired-helical filament (PHF) tau phosphorylation sites, Ser202 and Ser262 (Fig. 1A). Western analysis and densitometry, quantification of the phosphorylated tau revealed that overexpression of wild-type PTEN slightly but significantly decreased the level of Ser214 phospho-tau to 80% compared to vector transfected control (Fig. 1). The levels of phospho-tau at the other 6 examined sites did not show significant changes in the presence of wild-type PTEN compared to control (Fig 1B).

Dramatic changes in the levels of phospho-tau were observed when the FTDP-17 mutant tau was cotransfected with the catalyst activity null mutant PTEN. The levels of Thr212 and Ser214 phospho-tau were significantly increased by approximately 30% and 60%, respectively (Fig. 1B). Although Thr212 and Ser214 can also be phosphorylated by other kinases, including MAPK and PKC, besides Akt, the observation that the mutant tau phosphorylation at MAPK and PKC sites, such as Ser199, Ser202 and Ser262, did not exhibit any significant change in the presence of either wild-type or the mutant PTEN. This suggests that Akt, rather than MAPK and PKC, plays an important role in the mutant tau phosphorylation, and this effect can be regulated by PTEN activity.

### Overexpression of PTEN affects the FTDP-17 mutant tau aggregation and cellular distribution

**Figure 2:**
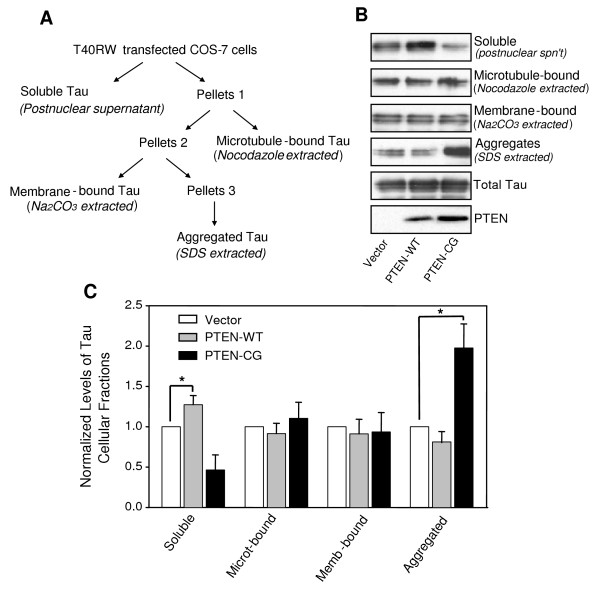
PTEN affects cellular distribution and binding to microtubule of the FTDP-17 mutant tau. A. The fractionation scheme used to resolve different cellular pools of tau. Total membrane and cytosolic fractions were prepared. The tau fraction sedimenting with total membranes (Pellet 1) was treated with nocodazole to solubilize microtubule-associated tau (microtubule-bound tau). The remaining membrane-bound/aggregated tau (Pellet 2) was treated with Na_2_CO_3 _to extract membrane-associated tau (membrane-bound tau). The remaining tau aggregates (Pellet 3) were dissolved by SDS. B. Western blot analysis of expression of PTEN and of tau in various celluar fractions. C. Quantification analysis. Data represent mean ± SE, *n *= 3, *p < 0.02.

It has been generally believed that hyperphosphorylation of tau can lead to tau aggregation. Given PTEN's effects on mutant tau's phosphorylation, we then asked if ectopic expression of wild-type and mutant PTEN can lead to changes of mutant tau aggregation and cellular distribution/partitioning. First, we determined cellular distribution of the mutant tau by fractionating the cells cotransfected with the FTDP-17 mutant tau, T40RW, and either wild-type PTEN, a catalytic activity dead PTEN mutant or vector (control). The transfected mutant tau in various fractions including cytosolic soluble tau, microtubule-bound tau, membrane-bound tau and SDS-soluble aggregated tau was detected by Western blots (Fig. 2A). In the presence of wild-type PTEN, there was about 20% more soluble tau in the cytosol compared to those in the control cells. Correlated with the increase of soluble tau, overexpression of wild-type PTEN decreased the level of the aggregated mutant tau by approximately 20%, indicating a shift between the pools of free tau and aggregated tau caused by PTEN. Similar to what happened in tau phosphorylation with the ectopic expression of the mutant PTEN, a significant shift from the cytosolic free tau to the aggregated tau was observed when the mutant PTEN was expressed: the amount of aggregated tau was nearly doubled while the soluble fraction of the mutant tau was decreased by 50% (Fig. 2).

**Figure 3:**
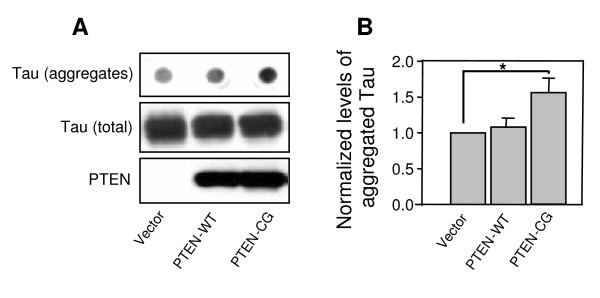
PTEN affects aggregation of the mutant tau. A. COS-7 cells transiently overexpressing the FTDP-17 mutant tau were transfected with PTEN-WT, PTEN-CG or control vectors. SDS-insoluble aggregated tau proteins were detected by using a filter-trap/immunoblotting assay. The transfected PTEN and total tau proteins (as indicated in middle and lower panels) were detected by Western blots from same amounts of lysates as in filter assays. B. The amounts of aggregated tau were quantified by densitometry and normalized against the amount of total tau. Error bars indicate means ± SE, *n *= 3, *p < 0.02 compared to the vector transfection control.

Using filter/trap assays, we isolated and quantified the amounts of SDS-insoluble tau aggregates from the mutant tau and PTEN cotransfected cells (Fig. 3A). After Western blots and densitometry, we found that the overexpression of wild-type PTEN did not significantly change the amount of insoluble tau aggregates compared to control. On the other hand, the level of the detergent resistant mutant tau aggregates was increased more than 50% in the cells cotransfected with the mutant PTEN compared to the control vector. These results, together with the changes of tau cellular distribution upon overexpression of PTEN, suggest that PTEN plays a role in mutant tau pathophysiological functions, likely through PTEN's regulatory effects on tau phosphorylation.

### Overexpression of the mutant PTEN caused formation of aggregates of the mutant tau in cells

**Figure 4:**
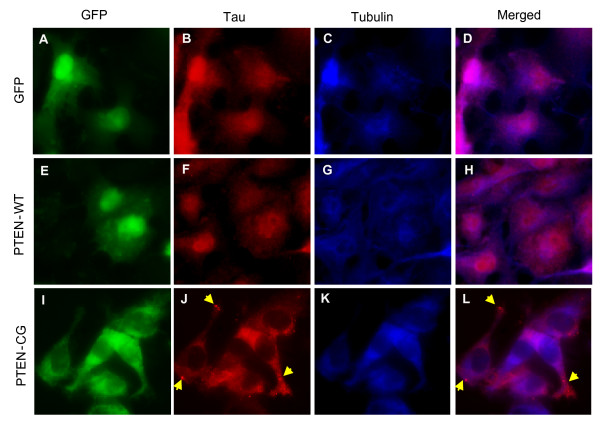
The phosphatase activity null PTEN leads to formation of visible tau aggregates in cells. COS-7 cells stably expressing the FTDP-17 mutant tau were transfected with pIRES-EGFP (A-D), pIRES-EGFP-PTENwt (E-H) or pIRES-EGFP-PTENcg (I-L). Expression of EGFP control (A), PTEN-WT (E) or PTEN-CG (I) was visualized based on the EGFP fluorescence. Cells were further immunostained to detect tau (B,F,J) and α-tubulin (C,G,K). Fluorescence micrographs were visualized and recorded by fluorescence microscope. D, H, and L are merged images of tau and α-tubulin immunostaining.

The observation that the significant increase of mutant tau aggregates in the presence of mutant PTEN led us to hypothesize that a mutation in PTEN may cause visible tau aggregates in cells. To test the hypothesis, COS-7 cells stably expressing T40RW tau were transfected with pIRES-EGFP-PtenWT, pIRES-EGFP-PtenCG or pIRES-EGFP as a control, and immunostained with anti-tau and anti-tubulin antibody. The expression of PTENs was represented by the expression of EGFP (Fig. 4A,E,I). The tau immunofluorescence was shown to only partially overlap with that of microtubules (Fig. 4D,H,L), suggesting a defect in microtubule binding of this mutant tau. The overexpression of wild-type PTEN did not change the cellular localization of the mutant tau or the interaction between the mutant tau and microtubules (Fig. 4H). On the other hand, upon overexpression of the mutant PTEN, we observed aggregates of the mutant tau in the cytosol (Fig. 4J,L), although the nature of the aggregates in how they resemble the NFTs remains to be determined. In addition, the reduced immunofluoresent colocalization between the mutant tau and microtubules indicated an impaired interaction between the two. Furthermore, we observed an abnormal pattern of tau immunostaining (Fig. 4J) and less-organized microtubule structures (Fig. 4K) in the mutant PTEN transfected cells compared to those in control vector and wild-type PTEN transfected cells. Given that the expression of the mutant PTEN alone in the cells did not cause disorganization of the microtubules (data not shown), the observed changes in microtubules in the mutant tau transfected cells are likely due to the formation of the mutant tau aggregates.

## Discussion

In a previous study, we found tumor suppressor PTEN regulates tau phosphorylation at multiple sites and affects tau aggregation and binding to microtubules. To further explore the role of PTEN in the pathogenesis of tauopathies, we examined phosphorylation of an FTDP-17 mutant tau in the presence of wild-type or the catalyst activity null mutant PTEN. Similar to what happened to wild-type tau [43], overexpression of PTEN (wild-type and the mutant) caused changes in tau phosphorylation most significantly at Akt sites, Thr212 and Ser214, suggesting that PTEN-regulated PI3K signaling also plays a role in phosphorylation of pathological tau mutants. It has been known that Ser214 is one of the major tau phosphorylation sites in NFTs whose phosphorylation interferes with the tau-microtubule interaction *in vitro *[50]. Together with the previous observation that tau is heavily phosphorylated at Ser214 in NFTs concomitant with decreased levels of PTEN in AD brains [25,43], our current results support the notion that Ser214 phosphorylation may be a crucial factor contributing to tauopathies, which can be affected by PTEN through the PI3K signaling pathway. However, since PTEN-modulated PI3K signaling also regulates other tau kinases besides Akt, the possibility that PTEN can affect tau phosphorylation at other sites through different mechanisms requires further investigation. In addition, since PTEN may exert its cellular functions independent of the PIP3 signaling pathway, e.g., inhibiting phosphorylation of transcription factor ETS-2 through MAPK [51], it remains possible that PTEN may affect the pathogenesis of tauopathy by a mechanism other than regulating the phosphorylation status of tau, such as by affecting tau ubiquitination and degradation.

We have previously shown that PTEN affects phosphorylation of wild-type tau at multiple sites. Here we demonstrate that PTEN affects the mutant tau most significantly at the Akt sites. This difference is likely due to the changes in the biochemical properties of tau caused by the missense FTDP-17 mutation, which may reflect the conformational/structural changes of the mutant tau, which could alter the accessibility of the mutant tau to the tau kinases and accelerate pathogenesis of tauopathy.

It has been shown that the FTDP-17 mutant tau proteins form filaments in transgenic mouse brains [52-54], and the tau filaments are stained by the AT100 antibody that detects phospho-tau at Ser214 and Thr212 [53,55], suggesting the mutant tau is hyperphosphorylated at the Akt sites. However, it has not been clarified why FTDP-17 mutant tau proteins fail to form aggregates and exhibit less phosphorylation at certain sites compared to wild-type tau in cultured cells [44,45,56-58]. In this study, we were able to detect tau aggregates in the cells coexpressing the mutant tau and the phosphatase activity null PTEN, resembling the *in vivo *observations. Our data suggested that abnormally upregulated PI3K signaling can forcefully increase tau phosphorylation at the two Akt sites that may play a key role in the pathogenesis of tauopathies, a notion that is supported by the observation that a higher Akt activity and loss of PTEN are indeed found in postmortem AD brains [25,43].

## Conclusion

In this study, we demonstrate that ectopic expression of wild-type or the phosphatase activity null mutant tumor suppressor PTEN can affect the FTDP-17 tau phosphorylation at important PHF sites to regulate tau's microtubule-binding function and aggregation. Our data suggest that mutations in *Pten *or deficiency in its phosphatase activity may lead to pathogenesis of tauopathies. In addition, our findings provide additional support for the link between the PI3K pro-survival signaling pathway and tauopathy in neurodegeneration, and potentially assign PTEN as a potential therapeutic target for AD.

## Methods

### Constructs

Human wild-type and mutant *Pten *cDNAs were subcloned into pIRES-EGFP (Invitrogen, Carlsbad, CA) to generate pIRES-*Pten *expression vectors. Specifically, the 1.2 kb *Pten *cDNA fragments were cut and collected from pEF-PtenWT and pEF-PtenCG (gifts from Dr. Hong Wu, UCLA) using *Eco*RI/*Bam*HI sites. The fragments were then ligated to *Eco*RI/*Bam*HI digested pIRES-EGFP to produce pIRES-EGFP-PtenWT and pIRES-EGFP-PtenCG.

### Cell cultures and transfection

COS-7 cells were maintained in DMEM medium supplemented with 10% FBS and antibiotics. Cells were first transfected with the mutant tau (T40RW) and equally split, followed by a second transfection with either wild-type PTEN or the lipid phosphatase null mutant PTEN (PTEN CG), using lipofectamine (Invitrogen). In some experiments, COS-7 cells stably expressing the FTDP-17 mutant tau (T40RW) were cultured on coverslips, and then transfected with pIRES-EGFP-PtenWT or pIRES-EGFP-PtenCG.

### Western blotting

To analyze phospho-tau, cells were homogenized in a lysis buffer containing 10 mM Tris/Cl, pH 7.4, 150 mM NaCl, 5 mM EDTA, 5 mM EGTA, 50 mM NaF, 1 mM Na_3_VOF_3_, 5 mM DTT, 1% NP-40 and a cocktail of protease inhibitors. Cell lysates were collected after brief sonication and centrifugation at 18,000 × *g*. Equal amounts of lysate samples were then subjected to SDS-PAGE. Proteins were transferred to PVDF membranes and probed with anti-tau antibodies: H150 (1:1000; Santa Cruz Biotechnology, Santa Cruz, CA), pS214 (1:1000; Biosource, Carlsbad, CA), pS199 (1:1000; Biosource), pT212 (1:1000; Biosource), pS396 (1:1000; Biosource), pS202 (1:1000; Biosource), pS262 (1:1000; Biosource) and pT205 (1:500; Biosource). PTEN proteins were detected using mouse anti-PTEN antibody (1:1000; Cell signaling, Danvers, MA). Tubulin was detected using anti-α-tubulin antibody (1:10000; Sigma, St. Louis, MO). The membranes were incubated with peroxidase-labeled secondary antibodies, and signals were visualized using ECL. In some experiments, Western blots were scanned and protein bands were quantified using Scion Image software.

### Fractionation of transfected COS-7 Cells

COS-7 cells were cotransfected with the mutant human tau and either wild-type, the mutant human *Pten*, or pcDNA control. Cells were fractioned as previously described with modifications [43,59]. Specifically, cells were harvested 48 h after transfection and homogenized in breaking buffer (0.25 M sucrose/10 mM Hepes, pH 7.2/1 mM MgOAc_2_/protease inhibitors mixture) by using a stainless steel ball-bearing homogenizer (18-μm clearance). Cytosol was prepared from postnuclear supernatant by ultracentrifugation for 1 h at 190,000 × *g*. The resulting membrane pellet was resuspended and incubated on ice for 30 min with 5 μM nocodazole, followed by ultracentrifugation for 1 h at 190,000 × *g *to produce post-nocodazole supernatants containing microtubule-associated tau. The generated pellets containing both membrane-associated and aggregated tau were further extracted using 100 mM sodium carbonate buffer (pH 11.5) at 4°C for 30 min. The post-Na_2_CO_3 _pellets were prepared by ultracentrifugation at 190,000 × *g *for 1 h and washed with 1% SDS to produce a fraction containing tau aggregates. Aliquots containing equal amounts of protein were analyzed by SDS/PAGE-Western blotting for tau using H150. Western blotting results were quantified by densitometry to determine the tau level in each fraction.

### Filter/trap assays for tau aggregates

The filter/trap assays were performed as described previously with minor modification [43,59]. Specifically, COS-7 cells expressing the FTDP-17 mutant human tau were transfected with human wild-type *Pten*, the mutant *Pten *or pcDNA control. Cells were lysed in a buffer containing 0.5% Nonidet P-40/1 mM EDTA/50 mM Tris HCl, pH 8.0/120 mM NaCl/protease inhibitors mixture. After brief sonication, cell lysates were passed through a cellulose acetate membrane (0.2 μm; Bio-Rad, Hercules, CA) using Bio-Dot Microfiltration Apparatus (Bio-Rad) and washed three times with 1% SDS followed by immunoblotting using H150 antibody. Quantitative Western blot analyses were used to determine the level of tau aggregates in each sample.

### Immunocytochemistry

To stain tau and tubulin in pIRES-EGFP-Pten transfected COS-7 cells that stably express the mutant human tau (T40RW), cells on coverslips were fixed in 4% paraformadelhyde (PFA)/PBS for 15 min followed by washing with PBS 5 times at 5 min each. Cells were then permeabilized with 0.1% Triton X-100 in PBS for 10 min before blocking with 5% BSA/PBS for 30 min. After washing with PBS, cells were incubated with anti-tau antibody, H150 (1:200; Santa Cruz Biotechnology) and anti-α-tubulin antibody (1:2000; Sigma) in 5% BSA/PBS for 2 hrs. Cells were then washed and incubated with 7-amino-4-methylcoumarin-3-acetic acid (AMCA)-conjugated anti-mouse IgG (1:300; Invitrogen) and Alexa Fluor 594-conjugated anti-rabbit IgG (Invitrogen; 1:300) for 1 h. The coverslips were then washed and mounted on slides. All procedures were performed at room temperature. Images were visualized and taken using deconvolution microscopy (Zeiss Axiovert 100 M).

## Competing interests

The author(s) declare that they have no competing interests.

## Authors' contributions

All authors read and approved the final manuscript. X.Z. and H.X. designed research; X.Z., Y.Z., S.L. and A.B. performed research; G.T. and Z.Z. contributed new reagents/analytic tools; X.Z., F.-F.L. and H.X. analyzed data; and X.Z. and H.X. wrote the paper.

## References

[B1] Trojanowski JQ, Lee VM (2000). "Fatal attractions" of proteins. A comprehensive hypothetical mechanism underlying Alzheimer's disease and other neurodegenerative disorders. Ann N Y Acad Sci.

[B2] Lee VM, Goedert M, Trojanowski JQ (2001). Neurodegenerative tauopathies. Annu Rev Neurosci.

[B3] Morsch R, Simon W, Coleman PD (1999). Neurons may live for decades with neurofibrillary tangles. J Neuropathol Exp Neurol.

[B4] Zheng H, Koo EH (2006). The amyloid precursor protein: beyond amyloid. Mol Neurodegeneration.

[B5] Vetrivel KS, Zhang Y, Xu H, Thinakaran G (2006). Pathological and physiological functions of presenilins. Mol Neurodegeneration.

[B6] Grundke-Iqbal I, Iqbal K, Tung YC, Quinlan M, Wisniewski HM, Binder LI (1986). Abnormal phosphorylation of the microtubule-associated protein tau (tau) in Alzheimer cytoskeletal pathology. Proc Natl Acad Sci U S A.

[B7] Weingarten MD, Lockwood AH, Hwo SY, Kirschner MW (1975). A protein factor essential for microtubule assembly. Proc Natl Acad Sci U S A.

[B8] Cleveland DW, Hwo SY, Kirschner MW (1977). Purification of tau, a microtubule-associated protein that induces assembly of microtubules from purified tubulin. J Mol Biol.

[B9] Cleveland DW, Hwo SY, Kirschner MW (1977). Physical and chemical properties of purified tau factor and the role of tau in microtubule assembly. J Mol Biol.

[B10] Baumann K, Mandelkow EM, Biernat J, Piwnica-Worms H, Mandelkow E (1993). Abnormal Alzheimer-like phosphorylation of tau-protein by cyclin-dependent kinases cdk2 and cdk5. FEBS Lett.

[B11] Hanger DP, Hughes K, Woodgett JR, Brion JP, Anderton BH (1992). Glycogen synthase kinase-3 induces Alzheimer's disease-like phosphorylation of tau: generation of paired helical filament epitopes and neuronal localisation of the kinase. Neurosci Lett.

[B12] Roder HM, Ingram VM (1991). Two novel kinases phosphorylate tau and the KSP site of heavy neurofilament subunits in high stoichiometric ratios. J Neurosci.

[B13] Roder HM, Eden PA, Ingram VM (1993). Brain protein kinase PK40erk converts TAU into a PHF-like form as found in Alzheimer's disease. Biochem Biophys Res Commun.

[B14] Robertson J, Loviny TL, Goedert M, Jakes R, Murray KJ, Anderton BH, Hanger DP (1993). Phosphorylation of tau by cyclic-AMP-dependent protein kinase. Dementia.

[B15] Baudier J, Cole RD (1987). Phosphorylation of tau proteins to a state like that in Alzheimer's brain is catalyzed by a calcium/calmodulin-dependent kinase and modulated by phospholipids. J Biol Chem.

[B16] Baudier J, Lee SH, Cole RD (1987). Separation of the different microtubule-associated tau protein species from bovine brain and their mode II phosphorylation by Ca2+/phospholipid-dependent protein kinase C. J Biol Chem.

[B17] Ksiezak-Reding H, Pyo HK, Feinstein B, Pasinetti GM (2003). Akt/PKB kinase phosphorylates separately Thr212 and Ser214 of tau protein in vitro. Biochim Biophys Acta.

[B18] Kopke E, Tung YC, Shaikh S, Alonso AC, Iqbal K, Grundke-Iqbal I (1993). Microtubule-associated protein tau. Abnormal phosphorylation of a non-paired helical filament pool in Alzheimer disease. J Biol Chem.

[B19] Kenessey A, Yen SH (1993). The extent of phosphorylation of fetal tau is comparable to that of PHF-tau from Alzheimer paired helical filaments. Brain Res.

[B20] Alonso AC, Zaidi T, Grundke-Iqbal I, Iqbal K (1994). Role of abnormally phosphorylated tau in the breakdown of microtubules in Alzheimer disease. Proc Natl Acad Sci U S A.

[B21] Alonso A, Zaidi T, Novak M, Grundke-Iqbal I, Iqbal K (2001). Hyperphosphorylation induces self-assembly of tau into tangles of paired helical filaments/straight filaments. Proc Natl Acad Sci U S A.

[B22] Hutton M, Lendon CL, Rizzu P, Baker M, Froelich S, Houlden H, Pickering-Brown S, Chakraverty S, Isaacs A, Grover A, Hackett J, Adamson J, Lincoln S, Dickson D, Davies P, Petersen RC, Stevens M, de Graaff E, Wauters E, van Baren J, Hillebrand M, Joosse M, Kwon JM, Nowotny P, Che LK, Norton J, Morris JC, Reed LA, Trojanowski J, Basun H, Lannfelt L, Neystat M, Fahn S, Dark F, Tannenberg T, Dodd PR, Hayward N, Kwok JB, Schofield PR, Andreadis A, Snowden J, Craufurd D, Neary D, Owen F, Oostra BA, Hardy J, Goate A, van Swieten J, Mann D, Lynch T, Heutink P (1998). Association of missense and 5'-splice-site mutations in tau with the inherited dementia FTDP-17. Nature.

[B23] Poorkaj P, Bird TD, Wijsman E, Nemens E, Garruto RM, Anderson L, Andreadis A, Wiederholt WC, Raskind M, Schellenberg GD (1998). Tau is a candidate gene for chromosome 17 frontotemporal dementia. Ann Neurol.

[B24] Spillantini MG, Murrell JR, Goedert M, Farlow MR, Klug A, Ghetti B (1998). Mutation in the tau gene in familial multiple system tauopathy with presenile dementia. Proc Natl Acad Sci U S A.

[B25] Griffin RJ, Moloney A, Kelliher M, Johnston JA, Ravid R, Dockery P, O'Connor R, O'Neill C (2005). Activation of Akt/PKB, increased phosphorylation of Akt substrates and loss and altered distribution of Akt and PTEN are features of Alzheimer's disease pathology. J Neurochem.

[B26] Li X, An WL, Alafuzoff I, Soininen H, Winblad B, Pei JJ (2004). Phosphorylated eukaryotic translation factor 4E is elevated in Alzheimer brain. Neuroreport.

[B27] An WL, Cowburn RF, Li L, Braak H, Alafuzoff I, Iqbal K, Iqbal IG, Winblad B, Pei JJ (2003). Up-regulation of phosphorylated/activated p70 S6 kinase and its relationship to neurofibrillary pathology in Alzheimer's disease. Am J Pathol.

[B28] Khurana V, Lu Y, Steinhilb ML, Oldham S, Shulman JM, Feany MB (2006). TOR-Mediated Cell-Cycle Activation Causes Neurodegeneration in a Drosophila Tauopathy Model. Curr Biol.

[B29] Stiles B, Groszer M, Wang S, Jiao J, Wu H (2004). PTENless means more. Dev Biol.

[B30] Sulis ML, Parsons R (2003). PTEN: from pathology to biology. Trends Cell Biol.

[B31] Stokoe D (2001). Pten. Curr Biol.

[B32] Li DM, Sun H (1997). TEP1, encoded by a candidate tumor suppressor locus, is a novel protein tyrosine phosphatase regulated by transforming growth factor beta. Cancer Res.

[B33] Li J, Yen C, Liaw D, Podsypanina K, Bose S, Wang SI, Puc J, Miliaresis C, Rodgers L, McCombie R, Bigner SH, Giovanella BC, Ittmann M, Tycko B, Hibshoosh H, Wigler MH, Parsons R (1997). PTEN, a putative protein tyrosine phosphatase gene mutated in human brain, breast, and prostate cancer. Science.

[B34] Steck PA, Pershouse MA, Jasser SA, Yung WK, Lin H, Ligon AH, Langford LA, Baumgard ML, Hattier T, Davis T, Frye C, Hu R, Swedlund B, Teng DH, Tavtigian SV (1997). Identification of a candidate tumour suppressor gene, MMAC1, at chromosome 10q23.3 that is mutated in multiple advanced cancers. Nat Genet.

[B35] Maehama T, Dixon JE (1998). The tumor suppressor, PTEN/MMAC1, dephosphorylates the lipid second messenger, phosphatidylinositol 3,4,5-trisphosphate. J Biol Chem.

[B36] Cantley LC, Neel BG (1999). New insights into tumor suppression: PTEN suppresses tumor formation by restraining the phosphoinositide 3-kinase/AKT pathway. Proc Natl Acad Sci U S A.

[B37] Di Cristofano A, Pesce B, Cordon-Cardo C, Pandolfi PP (1998). Pten is essential for embryonic development and tumour suppression. Nat Genet.

[B38] Podsypanina K, Ellenson LH, Nemes A, Gu J, Tamura M, Yamada KM, Cordon-Cardo C, Catoretti G, Fisher PE, Parsons R (1999). Mutation of Pten/Mmac1 in mice causes neoplasia in multiple organ systems. Proc Natl Acad Sci U S A.

[B39] Stambolic V, Suzuki A, de la Pompa JL, Brothers GM, Mirtsos C, Sasaki T, Ruland J, Penninger JM, Siderovski DP, Mak TW (1998). Negative regulation of PKB/Akt-dependent cell survival by the tumor suppressor PTEN. Cell.

[B40] Suzuki A, de la Pompa JL, Stambolic V, Elia AJ, Sasaki T, del Barco Barrantes I, Ho A, Wakeham A, Itie A, Khoo W, Fukumoto M, Mak TW (1998). High cancer susceptibility and embryonic lethality associated with mutation of the PTEN tumor suppressor gene in mice. Curr Biol.

[B41] Kwon CH, Zhu X, Zhang J, Knoop LL, Tharp R, Smeyne RJ, Eberhart CG, Burger PC, Baker SJ (2001). Pten regulates neuronal soma size: a mouse model of Lhermitte-Duclos disease. Nat Genet.

[B42] Fraser MM, Zhu X, Kwon CH, Uhlmann EJ, Gutmann DH, Baker SJ (2004). Pten loss causes hypertrophy and increased proliferation of astrocytes in vivo. Cancer Res.

[B43] Zhang X, Li F, Bulloj A, Zhang Y, Tong G, Zhang Z, Liao F, Xu H (2006). Tumor suppressor PTEN affects tau phosphorylation, aggregation and binding to microtubules. FASEB J.

[B44] Perez M, Lim F, Arrasate M, Avila J (2000). The FTDP-17-linked mutation R406W abolishes the interaction of phosphorylated tau with microtubules. J Neurochem.

[B45] Vogelsberg-Ragaglia V, Bruce J, Richter-Landsberg C, Zhang B, Hong M, Trojanowski JQ, Lee VM (2000). Distinct FTDP-17 missense mutations in tau produce tau aggregates and other pathological phenotypes in transfected CHO cells. Mol Biol Cell.

[B46] Reed LA, Grabowski TJ, Schmidt ML, Morris JC, Goate A, Solodkin A, Van Hoesen GW, Schelper RL, Talbot CJ, Wragg MA, Trojanowski JQ (1997). Autosomal dominant dementia with widespread neurofibrillary tangles. Ann Neurol.

[B47] Myers MP, Pass I, Batty IH, Van der Kaay J, Stolarov JP, Hemmings BA, Wigler MH, Downes CP, Tonks NK (1998). The lipid phosphatase activity of PTEN is critical for its tumor supressor function. Proc Natl Acad Sci U S A.

[B48] Wang X, Gjorloff-Wingren A, Saxena M, Pathan N, Reed JC, Mustelin T (2000). The tumor suppressor PTEN regulates T cell survival and antigen receptor signaling by acting as a phosphatidylinositol 3-phosphatase. J Immunol.

[B49] Liu F, Grundke-Iqbal I, Iqbal K, Gong CX (2005). Contributions of protein phosphatases PP1, PP2A, PP2B and PP5 to the regulation of tau phosphorylation. Eur J Neurosci.

[B50] Illenberger S, Zheng-Fischhofer Q, Preuss U, Stamer K, Baumann K, Trinczek B, Biernat J, Godemann R, Mandelkow EM, Mandelkow E (1998). The endogenous and cell cycle-dependent phosphorylation of tau protein in living cells: implications for Alzheimer's disease. Mol Biol Cell.

[B51] Weng LP, Brown JL, Baker KM, Ostrowski MC, Eng C (2002). PTEN blocks insulin-mediated ETS-2 phosphorylation through MAP kinase, independently of the phosphoinositide 3-kinase pathway. Hum Mol Genet.

[B52] Tatebayashi Y, Miyasaka T, Chui DH, Akagi T, Mishima K, Iwasaki K, Fujiwara M, Tanemura K, Murayama M, Ishiguro K, Planel E, Sato S, Hashikawa T, Takashima A (2002). Tau filament formation and associative memory deficit in aged mice expressing mutant (R406W) human tau. Proc Natl Acad Sci U S A.

[B53] Gotz J, Chen F, Barmettler R, Nitsch RM (2001). Tau filament formation in transgenic mice expressing P301L tau. J Biol Chem.

[B54] Lim F, Hernandez F, Lucas JJ, Gomez-Ramos P, Moran MA, Avila J (2001). FTDP-17 mutations in tau transgenic mice provoke lysosomal abnormalities and Tau filaments in forebrain. Mol Cell Neurosci.

[B55] Gotz J, Tolnay M, Barmettler R, Chen F, Probst A, Nitsch RM (2001). Oligodendroglial tau filament formation in transgenic mice expressing G272V tau. Eur J Neurosci.

[B56] Matsumura N, Yamazaki T, Ihara Y (1999). Stable expression in Chinese hamster ovary cells of mutated tau genes causing frontotemporal dementia and parkinsonism linked to chromosome 17 (FTDP-17). Am J Pathol.

[B57] Mack TG, Dayanandan R, Van Slegtenhorst M, Whone A, Hutton M, Lovestone S, Anderton BH (2001). Tau proteins with frontotemporal dementia-17 mutations have both altered expression levels and phosphorylation profiles in differentiated neuroblastoma cells. Neuroscience.

[B58] DeTure M, Ko LW, Easson C, Yen SH (2002). Tau assembly in inducible transfectants expressing wild-type or FTDP-17 tau. Am J Pathol.

[B59] Dou F, Netzer WJ, Tanemura K, Li F, Hartl FU, Takashima A, Gouras GK, Greengard P, Xu H (2003). Chaperones increase association of tau protein with microtubules. Proc Natl Acad Sci U S A.

